# A Context-Specific Digital Alcohol Brief Intervention in Symptomatic Breast Clinics (Abreast of Health): Development and Usability Study

**DOI:** 10.2196/14580

**Published:** 2020-01-24

**Authors:** Julia M A Sinclair, Peter F Dutey-Magni, Annie S Anderson, Janis Baird, Mary E Barker, Ramsey I Cutress, Eileen F S Kaner, Mark McCann, Caspian K Priest, Ellen R Copson

**Affiliations:** 1 Faculty of Medicine University of Southampton Southampton United Kingdom; 2 Institute of Health Informatics University College London London United Kingdom; 3 Centre for Research into Cancer Prevention and Screening Division of Population Health & Genomics University of Dundee Medical School Dundee United Kingdom; 4 Medical Research Council Lifecourse Epidemiology Unit University of Southampton Southampton United Kingdom; 5 National Institute of Health Research Southampton Biomedical Research Centre University Hospital Southampton NHS Foundation Trust Southampton United Kingdom; 6 Institute of Health and Society Newcastle University Newcastle United Kingdom; 7 Medical Research Council/Scottish Government Chief Scientist Office Social and Public Health Sciences Unit University of Glasgow Glasgow United Kingdom

**Keywords:** cancer, information seeking behavior, health risk behaviors, secondary prevention, alcohol drinking, health knowledge, attitudes, practice, health promotion, health literacy

## Abstract

**Background:**

Potentially modifiable risk factors account for approximately 23% of breast cancer cases. In the United Kingdom, alcohol consumption alone is held responsible for 8% to 10% of cases diagnosed every year. Symptomatic breast clinics focus on early detection and treatment, but they also offer scope for delivery of low-cost lifestyle interventions to encourage a cancer prevention culture within the cancer care system. Careful development work is required to effectively translate such interventions to novel settings.

**Objective:**

The aim of this study was to develop a theory of change and delivery mechanism for a context-specific alcohol and lifestyle brief intervention aimed at women attending screening and symptomatic breast clinics.

**Methods:**

A formative study combined evidence reviews, analysis of mixed method data, and user experience research to develop an intervention model, following the 6 Steps in Quality Intervention Development (6SQuID) framework.

**Results:**

A Web app focused on improving awareness, encouraging self-monitoring, and reframing alcohol reduction as a positive choice to improve health was found to be acceptable to women. Accessing this in the clinic waiting area on a tablet computer was shown to be feasible. An important facilitator for change may be the heightened readiness to learn associated with a salient health visit (a teachable moment). Women may have increased motivation to change if they can develop a belief in their capability to monitor and, if necessary, reduce their alcohol consumption.

**Conclusions:**

Using the 6SQuID framework supported the prototyping and maximized acceptability and feasibility of an alcohol brief intervention for women attending symptomatic breast clinics, regardless of their level of alcohol consumption.

## Introduction

### Background

Breast cancer is the most common type of cancer worldwide, and its incidence is rising [1]. The World Health Organization considers that sufficient knowledge is available to prevent 30% to 50% of cancer cases globally and that “prevention offers the most cost-effective long-term strategy for the control of cancer” [2]. In the United Kingdom, the proportion of breast cancer cases attributable to lifestyle factors is as follows: insufficient physical activity—2%, overweight or obesity—8%, and alcohol consumption—between 8% [3] and 10% [4]. Alcohol increases the risk of breast cancer in a dose-dependent fashion, even from low-risk drinking levels, with an estimated relative risk of 1.09 for 10 g/day [5]. Observational evidence shows that alcohol consumption may also increase the risk of recurrence of breast cancer in survivors [6,7]. New UK clinical guidelines advise this group to observe an upper limit of 5 units per week [8].

Systematic reviews of alcohol interventions indicate that, outside of regulatory interventions, alcohol brief interventions (ABIs) demonstrate the greatest effectiveness and cost effectiveness [9-11], with small reductions in alcohol consumption (20g/week) that can be sustained for at least a year [12,13]. Despite this, ABIs remain relatively underutilized across health care systems. In England, fewer than 7% of “increased-risk” drinkers recall receiving advice from their general practitioner on their alcohol consumption in the past year, compared with 50% of smokers who recalled receiving tobacco cessation advice [14].

The use of “teachable moments” is increasingly advocated to encourage modification of lifestyle determinants of cancers [15-17], but more research is required as to how best to situate health prevention interventions into current health systems. In England, over 540,000 women annually attend UK National Health Service (NHS) symptomatic breast clinics [18] as part of a rapid referral (2-week wait) system to prevent delay in diagnosis. Because fewer than 8% of women attending are found to have breast cancer [19], and health promotion information is not offered to those without a diagnosis, in prevention terms, the majority do not currently benefit from attending the clinic.

### Objectives

Previous research has criticized the premature trialing of ABIs in new environments, with recommendations that “applications of brief intervention to novel settings should begin with foundational research and developmental studies” [20]. This paper describes the development of a context-specific ABI aimed at women attending symptomatic breast clinics, using the 6 Steps in Quality Intervention Development (6SQuID) [21], a framework commonly employed in the development of public health interventions.

## Methods

### Framework

The 6SQuID framework [21] is intended to improve the design of public health interventions and, consequently, their effectiveness. This study synthesized information from 4 sources of data (reviews, empirical data from the target population, theory and concept mapping, and iterative content appraisal and design) to complete these steps in the breast health setting (Table 1).

**Table 1:** The 6 steps of the Quality Intervention Development framework as applied in the development of Abreast of Health (adapted from the study by Wight et al [21]).

Step and data provenance		Methods
**1. Define and understand the problem and its causes**		
	Attitudes literature (E^a^)	Scoping review
	Scoping study [22] (E)	Scoping review
**2. Clarify which causal or contextual factors are malleable and have greatest scope for change**		
	Risk attitude literature (E)	Scoping review and theory mapping
	Scoping study [22] (E)	Scoping review and theory mapping
	Review of existing apps (N^b^)	Scoping review and theory mapping
**3. Identify how to bring about change: the change mechanism**		
	Behavior change technique review (E)	Theory and concept mapping
**4. Identify how to deliver the change mechanism**		
	Behavior change technique review (E)	Concept mapping
	User testing (N)	Agile prototyping
**5. Test and refine on small scale**		
	User testing (N)	“Think aloud” and “teach me back” cognitive interviewing
**6. Collect sufficient evidence of effectiveness to justify rigorous evaluation/implementation**		
	To be addressed in future publication (N)	—^c^

^a^(E): existing data from the public domain.

^b^(N): new data generated during this study.

^c^Not applicable.

### Reviews

The academic and gray literature were reviewed iteratively in 3 different areas relevant to the intervention (Multimedia Appendix 1):

1. Knowledge and social attitudes to alcohol among women (particularly in the United Kingdom) and among health care staff—this included information on knowledge of alcohol volumes, effect of alcohol on health, and confidence in managing alcohol-related health risks.
2. Knowledge and social attitudes in relation to modifiable risk factors for cancer—particular attention was paid to interaction with social determinants of health, including health literacy, socioeconomic status, and social deprivation.
3. Findings from existing reviews on behavior change mechanisms and techniques for reducing alcohol consumption—in addition to reviews from the Cochrane library, we focused on systematic and narrative reviews of features of digitally delivered ABIs [23-27].

### Mixed Method Study With the Target Population

A mixed method study was undertaken to complement evidence from the literature reviews, with data from the target environment: symptomatic breast clinics and an NHS Breast Screening Programme unit in Southampton, United Kingdom. A total of 205 women attending appointments were recruited to take part in (1) a survey of knowledge of risk factors for breast cancer and alcohol beverage content and (2) 5 focus groups. Moreover, 33 health professionals took part in a similar survey, of whom 8 also participated in semistructured interviews. The full detail is reported separately [22], but it will be referred to here as part of the intervention development process.

### Theory and Concept Mapping

As part of 6SQuID steps 3 to 4, relevant theories and behavior change constructs were reviewed and mapped onto harmonized constructs from 2 systematic collations of health psychology theories commonly used in meta-analyses. These were (1) the 26 mechanisms of action [28] consolidating and extending the preexisting Theoretical Domains Framework [29] and (2) the 93 behavior change techniques (BCTs) from the BCT Taxonomy v1.1 [30].

### Iterative Content Appraisal and Design

The structure and content (both textual and visual) of the intervention prototype were designed by JMAS, PDM, and CKP in an Agile approach [31] between December 2016 and April 2017. This method relied on rapid prototyping and testing of small components using short cycles:

1. The research team scoped, reviewed, and appraised existing alcohol information leaflets, Web, and mobile phone apps. This involved mapping BCTs and appraising the language, tone, and focus of different approaches to consolidate a view of the most adapted content. A particular focus was placed on identifying features that were deemed difficult to understand, that were insufficiently relevant, or that could be perceived by some women as scary and/or judgmental. Similarly, features that appeared most helpful at implementing target mechanisms of change were also noted.
2. A total of 10 women recruited from symptomatic breast clinics were invited to test and comment on a range of existing health apps in 1 focus group, adding to findings from the team’s own analysis.
3. The research team sketched the visual layout of small components of the intervention.
4. Immediate comments and reactions on early versions of wording and visual features of these components were invited from 161 women recruited from symptomatic breast clinics. Participants took part in face-to-face cognitive interviews, which invited them to “think aloud” and “teach back” information gathered while testing the prototype to the researcher [32,33].

New findings were discussed by the research team on a weekly basis, setting objectives for the next data collection cycle the following week. Conclusions from these activities were mapped to a particular component of the emerging prototype intervention and recorded on a Kanban board (using the Trello software) [34] together with lists of actions, to incorporate them in the design work at every iteration of the weekly cycles.

All participants were recruited from the women attending the symptomatic breast clinics at Southampton General Hospital on referral from their primary care physician. All participants were approached in the waiting room, and having given consent, they either participated at that time and/or agreed to take part in a focus group/testing session at a later date. Activities (2) and (3) above were approved by Health Research Authority Research Ethics Committees as part of 2 independent studies (references: 17/LO/0953 and 18/SC/0120).

## Results

### Steps 1 to 2: Causal and Contextual Factors of the Target Problem

Having identified alcohol consumption as a potentially modifiable lifestyle cause of breast cancer, we undertook a broad review of underpinning factors (Multimedia Appendix 1). Table 2 gives a thematic summary of dominant themes of social and psychological determinants of knowledge, attitudes, and behavior around alcohol consumption.

Key findings were that although 60% to 72% of women attending breast screening appointments or symptomatic breast clinics drink alcohol, only 20% of women were aware that it was a risk factor for breast cancer [22,35-60]. Despite efforts from public campaigns informing the population of the effects of alcohol on long-term health, recent studies still demonstrate that the UK population recognizes these far less than the social harms of alcohol. This focus on risks associated with “binge” drinking (high-intensity, single-occasion alcohol use) can dim the awareness of the effects of consuming alcohol in lesser quantities across a sustained period. A recent UK–based qualitative study by Khadjesari et al [38] examined attitudes to alcohol and the government’s low-risk drinking guidelines (recommendation not to drink more than 14 (UK) units a week on a regular basis, maintaining several drink-free days per week) [37] among adults attending primary care facilities. The authors argued that the narrow public understanding of risks focused on the effects of high-intensity consumption of alcohol reduces the perceived relevance of low-risk drinking guidelines and contributes to participants’ belief that the 14-unit threshold is unnecessarily low.

From steps 1 to 2, we concluded that the greatest scope for change resides in increasing awareness of alcohol’s role in promoting chronic conditions such as cancer, even at low levels. This interacts with other behavioral predictors listed in Table 2, some of which are situated in the cancer context. For example, attitudes and beliefs such as cancer predeterminism and fatalism affect engagement with prevention behaviors [57,58] and the perceived relevance of information of lifestyle risk factors.

**Table 2:** Thematic summary of social and psychological determinants of knowledge, attitudes, and behavior around alcohol consumption.

Domain	Evidence
Knowledge: low alcohol literacy	Only 20% of women in breast clinics [22] identified alcohol consumption as a risk factor for breast cancer, a similar proportion as in the general population [35,36]. This lack of awareness is singled out as an obstacle in promoting low-risk drinking by the UK Chief Medical Officers [37]. Some common beliefs about alcohol and cancer are incorrect, for example: that alcohol only becomes a health risk in “problem drinkers” or people who are alcohol dependent; red wine being the only type of alcohol causing cancer; conversely, red wine/moderate alcohol intake being good for health; physical exercise mitigating the effects of heavy drinking [38].
Knowledge: low alcohol numeracy	Individuals do not always accurately recall the frequency, volume, and concentration of alcohol they drink [39,40]. Improving numeracy and encouraging monitoring of alcohol intake within primary care have been proposed by some [41,42] as a population prevention strategy.
Social role and identity of health professionals	In addition to lacking in time and relevant training on lifestyle interventions, health care staff may not believe it is part of their clinical role to discuss lifestyle factors in relation to modifiable risk factors for cancer [43-46]. Evidence also points to health professionals lacking in awareness of the causes of cancer, relevant lifestyle guidance, and the appropriate advice to give [43,47,48], and it points to lacking confidence that information will motivate women to change behaviors [43,47-49], sometimes hindered by the health care professionals’ own lifestyle choices [50]. Clinicians perceive a lack of patient interest in the subject [48] and tend to underestimate evidence on the fact that changing behaviors affect breast cancer risk [43,47-49].
Beliefs about capability and readiness to learn	Patients are more concerned by genetic determinants rather than modifiable risk factors for breast cancer [51]. Previous research has found some skepticism and defensiveness toward health promotion messages related to alcohol [52,53]. In some individuals, health literacy levels may be an obstacle to processing and making decisions based on the information given [54]. Many lack skill or confidence in taking practical steps to reduce alcohol consumption [54,55].
Health beliefs: cancer predeterminism and fatalism	A proportion of the population believes that incidence of cancer is purely down to “fate” or known genetic causes. “Cancer fatalism” is thought to have a negative impact on health behaviors, including screening uptake. Evidence suggests that it is more prevalent among women from black and minority ethnic backgrounds and that beliefs that cancer is predetermined are strongest among women: (1) born outside the United Kingdom, (2) whose main language is not English, or (3) exhibiting lower levels of health literacy [56]. Fatalistic beliefs are correlated with lifestyle [57], and these mediate the relationship between health literacy and information seeking [58].
Exposure to fear appeal messages	Alcohol and cancer are health themes in which public health campaigns have traditionally appealed to fear processes, seeking impact by evoking a strong emotional response. Alcohol harm reduction video advertisements, in particular, tend to have a negative emotional tone (74%) and focus on short-term risks (53%), with only 18% focusing on how to adapt lifestyle to improve long-term health [59]. This contributes to a subtext, which may trigger fear by association, even when unintended.
Perceived relevance of alcohol prevention	Generalist alcohol brief interventions are rarely tailored to individuals’ drinking behavior. We found that many leaflets contain messages and recommendations that are aimed at higher-risk drinkers; therefore, these are not relevant to many recipients’ level of alcohol consumption or lifestyles. These messages may, therefore, be easily dismissed by the majority of readers as irrelevant [38].

### Step 3: Mechanisms of Action

Beyond the need to increase knowledge of the long-term health effects of alcohol (commonly invoked as a necessary mechanism of action to promote behavior change) [28], attitudes toward the behavior and perceived susceptibility/vulnerability play a role. From existing reviews of behavior change mechanisms and techniques, we explored the role of emotions and perceived susceptibility/vulnerability in mediating or moderating alcohol behavior change.

The teachable moment model [61] posits that some health events facilitate behavior change by affecting subjects’ perception of personal risks, by evoking an affective response (such as a worry), which challenges their health-related beliefs to the point of promoting behavior change. However, this effect could be moderated by other processes in situations perceived as threats to life (eg, a potential cancer diagnosis). Under the assumption that a symptomatic breast referral raises the level of fear or perceived vulnerability, the extended parallel process model by Witte et al [62] anticipates one of two main responses: participants either accept related health messages (danger control processes) or reject them (fear control processes).

Danger control processes predict an enhanced “readiness to learn,” which we define as the propensity to absorb information on health risks, reflect on its meaning, and use it in relation to everyday lifestyle choices. An ABI could capitalize on danger control processes by establishing an association between alcohol and the risk of breast cancer and redirecting the individual’s attention toward achievable methods of reducing alcohol consumption.

Conversely, an ABI could fail by triggering fear control processes, by exacerbating fatalistic thoughts in women attending clinic who believe that cancer risk is largely predetermined and beyond their control. Such beliefs are known to be more prevalent in populations with limited health literacy [56]. If fear control processes dominate, recipients of the ABI may be inclined to discard lifestyle advice in an effort to manage or control their fear of cancer.

Data from our focus groups indicated that although fear control processes occur among women attending breast clinics (eg, “information overload,” avoidance of health literature), the desire to learn about modifiable risk factors is also present [61,63,64]. Studies by Anderson et al [65] have shown that the anxiety generated by a breast mammogram, far from constituting an obstacle to health promotion, can be used for opportunistic large-scale lifestyle interventions. Adapting the content of the intervention so as to minimize fear control processes is thus the main avenue to activating the potential efficacy of a teachable moment.

In addition to the findings from our reviews, qualitative evidence we collected [22,64] suggested that an intervention would need to enhance the perception that, out of all cancer risk factors, alcohol is one of the most easily modified, and it is necessary to emphasize the health and well-being *gains* of adopting and/or maintaining a lower level of alcohol consumption. Framing low-risk alcohol consumption levels in terms of “health gains” [66], using positive language, may be particularly important in the areas of cancer and alcohol use, where health promotion has been dominated by fear appeal techniques (eg, campaigns on missing the early signs of cancer or against drink driving). As individuals targeted by the proposed intervention will be influenced by their previous exposure to primarily fear-based messages, we specifically monitored the meaning early testers gave to health promotion messages embedded in the prototype intervention.

From step 3, we concluded that the intervention is most likely to succeed if it provides reassurance that alcohol is a controllable determinant of cancer and that it promotes positive benefits of limiting alcohol use for long-term health and well-being.

### Step 4: How to Deliver the Change Mechanism

Our previous work identified that the most feasible and scalable mode of delivering a lifestyle intervention in clinics was a Web app accessed by women in the clinic waiting area on a tablet computer [22]. In addition to circumventing the health care professional’s lack of time and confidence in delivering lifestyle brief interventions, preliminary user testing confirmed that electronic delivery was acceptable and brought advantages in terms of privacy.

Within the constraints set by a Web app, and with the help of the third review, we identified candidate BCTs to deliver the following mechanisms of action (see Table 3):

- Improving knowledge of the health benefits of low-risk drinking;
- Increasing skills in relation to estimating the alcohol content of beverages;
- Changing attitudes to, and beliefs about consequences of, alcohol consumption; and
- Capitalizing on perceived susceptibility/vulnerability heightened by the symptomatic breast clinic attendance to increase motivation while emphasizing personal control and belief in the capability to reduce cancer risk.

The 4 BCTs employed with the highest degree of fidelity across the prototype were as follows: provision of information on health consequences of alcohol, feedback on behavior, discrepancy between current behavior and goals, and social comparison. Other techniques, for example, self-monitoring or instructions on how to perform the behavior, informed the design of prompts or suggestions deeper in the application interface available to those who were interested in exploring them rather than being delivered procedurally by the interface to all users.

**Table 3:** Behavior change techniques and features identified for prototyping.

Behavior change techniques (taxonomy number)	Prototype features
Information about health consequences (5.1)	Information on alcohol’s dose-response association with breast cancer and the absence of a safe threshold Information on the proportion of breast cancer cases attributable to alcohol in the United Kingdom Information about benefits of low-risk drinking beyond lower risks of breast cancer (other types of cancers, mental health, dementia, liver, etc) “Myth busting” quiz on risk factors for breast cancer
Feedback on behavior (2.2) and discrepancy between current behavior and goal (1.6)	Assessment of current alcohol consumption in units per week Personalized feedback based on the UK Chief Medical Officers’ low-risk drinking guidance [37] Automated suggestion of 1 of 3 goals in line with the same guidance [37], as a function of the current pattern of alcohol consumption measured by the app: (1) low-risk drinkers: maintain current low-risk drinking; (2) low-frequency and high-intensity drinkers: have no more than 5 units of alcohol in any 1 day; (3) increased-risk drinkers: reduce alcohol consumption by a specified number of units per week (to reduce their consumption to under 14 units/week), with equivalent amount presented in number of wine glasses
Social comparison (6.2)	Personalized feedback of current alcohol consumption compared with (1) other women in England and (2) other women in the clinic
Framing/reframing (13.2)	Frame alcohol as an easily controllable risk factor for breast cancer Focus messages on risk reduction by changing behavior rather than risk promotion by current behavior (gain framing) Frame alcohol as any other health risk factor by embedding alcohol within broader information on lifestyle determinants of health: physical activity, diet, and weight Offer ways to reduce alcohol consumption and promote them as simple and easy steps Emphasize choice, presenting change as an easy option, with advice on how to cut down
Self-monitoring of behavior (2.3)	“Top tips”: recommend keeping a diary of alcohol intake with a mobile phone app (hyperlink to National Health Service drinks tracker app) or a paper diary (hyperlink to a diary template)
Credible source (9.1)	“Myth busting” quiz challenging common misunderstandings on risk factors believed to promote breast cancer Breast Cancer Now charity logo and endorsement National Health Service branding of the app (requested by women, to be implemented subject to relevant authorizations) Delivery of the intervention within the clinic waiting room, endorsement by health care staff
Instruction on how to perform a behavior (4.1); behavior substitution; and problem solving (1.2)	“Top tips”: examples of techniques to reduce alcohol consumption on social occasions, by setting goals, self-monitoring, and involving relatives “Top tips”: advice on choosing beverages with lower alcohol content and/or smaller volume; alternating drinks with glasses of water Drink calculator: information on beverage sizes and alcohol content in UK units Hyperlinks to further resources: drinking diary template, Public Health England drink tracker app, “Soberistas,” and “Club Soda”

#### Information About Health Consequences (Behavior Change Technique 5.1)

Information related to consequences for the risk of breast cancer was designed to convey the dose-dependent nature of the association between alcohol and breast carcinogenesis, emphasizing that no “safe” threshold exists for alcohol consumption in relation to breast cancer risk. The material designed by the team is adapted from an existing information leaflet [67] developed by a partner charity (Breast Cancer Now) on the basis of extensive qualitative research.

#### Feedback on Behavior (Behavior Change Technique 2.2), Discrepancy Between Current Behavior and Goal (Behavior Change Technique 1.6), and Social Comparison (Behavior Change Technique 6.2)

As women are often unsure about their alcohol risk levels (Table 2), study participants indicated that personalized feedback needed to be the first step of the intervention. Therefore, we assessed a range of questionnaires to assess current alcohol consumption or risk level. Testing of existing mobile phone and Web apps in the focus group confirmed that women wished to position themselves on a risk gradient to identify the scale of change they needed to undertake. We found that stratification tools that included items measuring social risks of alcohol were off-putting (eg, the items on injuries or feelings of guilt in the Alcohol Use Disorders Identification Test [68]). Such items triggered perceptions associated with substance “abuse,” which diverted attention from dose-dependent processes putting them at risk of chronic medical conditions. Therefore, we chose a short consumption-focused 3-item questionnaire, the Extended Alcohol Use Disorders Identification Test-consumption items (“Extended AUDIT-C”), and we are currently validating an algorithm that estimates average weekly alcohol consumption based on these 3 items.

#### Framing/Reframing of Alcohol (Behavior Change Technique 13.2)

The content of the intervention sought to reframe alcohol as one of the more controllable lifestyle risk factors for chronic illness (Table 3). We aimed to do the following:

- Offer a new perspective on low-risk drinking as a positive choice (gain framing) made to improve future health prospects
- Challenge binary stereotyping of alcohol use opposing “safe drinkers” and “alcoholics/boozers”; instead, represent the risks of drinking as a continuum. The language describing alcohol risks was kept as neutral as possible to adapt to a wide audience, and we excluded references to addiction or social harms of alcohol [38].

Some BCTs were potentially unhelpful in the context of the teachable moment within our target health settings because of their potential to trigger fear control reactions. In particular, we did not wish to enhance the *salience of health consequences of alcohol drinking* (BCT 5.2) or evoke *anticipated regret* (BCT 5.5) as the situational context of the breast clinic already made potential consequences of breast cancer tangible and memorable.

Finally, we identified other features likely to mediate the efficacy of the intervention, which required consideration as part of the iterative design and testing stage. As the usability of an electronic intervention is a predictor of engagement [69], we paid attention to women’s evaluation of its quality and discoverability (the extent to which women were able to find content on the app without being told it existed). We allowed the users to assess the alcohol content of their own preferred alcoholic drinks, and we sought to make “top tips” easy to navigate to enable participants to focus on specific information of interest to them.

### Step 5: Iterative Design, Testing, and Refining of a Prototype Intervention

Following a phase of testing, with cycles of refinement of the prototype with 161 women in clinics, the final prototype consisted of the following:

1. An initial assessment of alcohol consumption, smoking, height, and weight.
2. Personalized feedback on alcohol intake integrated with other risk factors: A feedback page presents the estimated number of units per week, and drinking risk level, assisted by a graphic visualizing alcohol risk levels based on the UK Chief Medical Officers’ guidance [37] (Multimedia Appendix 2). Individuals can compare their own drinking risk level with: the Department of Health low-risk drinking guidelines; drinking risk levels of women nationally; the proportion of other women attending the same clinic who drink at a similar risk level. To reduce stigma, this feedback is integrated with more succinct personalized feedback on benefits of not smoking; success rates of quit attempts; and ranges of healthy weights corresponding to the person’s height, with a button linking to health promotion content on physical activity and diet. The study participants improved the wording of the personalized feedback wherever it proved confusing or off-putting (eg, feedback aimed at low-frequency but high-intensity alcohol consumption was rephrased from “drinking large quantities” to “having no more than 5 units” on any single day).
3. An overview page linking to other health promotion information, including the following:
  - A myth-busting quiz testing knowledge on modifiable risk factors for breast cancer, including alcohol.
  - Information on the dose-response association between breast cancer and alcohol.
  - An interactive drink calculator providing alcohol units and calories of standard drinks as well as larger volumes (eg, bottles). This was refined to help participants add up, over any period, how many units of alcohol they may be consuming; how many kilocalories these drinks contain; food equivalents (in hamburgers and biscuits); and metabolic equivalents in minutes of tasks such as running, swimming, or housework.
  - Example goals for maintaining low-risk drinking or reducing alcohol consumption.
  - Specific information pages on the following: weight management, physical activity, diet, and smoking. A section on breast symptoms initially designed and tested was removed to refocus content on lifestyle promotion.

## Discussion

### Principal Findings

This study applied a rigorous intervention development framework, drawing on a suite of reviews of the risk factor literature, attitudes toward modifiable risk factors for cancer, and digital health interventions. We involved women attending breast clinics in the design, prototyping, and testing of a context-specific digital ABI in breast health settings with a potential to reach over 540,000 women per year in England alone, at very low costs, and where little information is currently provided in relation to modifiable risk factors for breast cancer. Coined as “teachable moments” in the cancer prevention literature [15], breast appointments constitute a privileged opportunity to raise awareness of potentially preventable causes of breast cancer. This assumes the provision of relevant, acceptable, and effective health promotion messages delivered with the highest level of fidelity.

The mechanisms of actions identified in this paper and our reviews of their evidence base suggest potential to achieve small reductions in alcohol consumption. Several moderators of the mechanisms of change for this intervention have been identified: acceptability to women, particularly those whose anxiety makes them potentially averse to health-related information; usability of the Web app delivering the intervention; and engagement with the subcomponents of the digital interface. The next phase of research will evaluate the feasibility, acceptability, and usability of the intervention in clinics with the target population and produce the necessary evidence on how to optimize the effect of such moderators.

### Comparison With Prior Work

The design of the proposed intervention differs from that of other digital ABIs, which focus either on student populations or longer-term engagement with mobile phone or Web apps [13,70]. In a clinical setting characterized by a high throughput and a narrow window for engagement, our development has focused on designing content that engages with the user as quickly as possible and is relevant to the widest range of women attending. This is a marked difference from other precedents in the United Kingdom such as Down Your Drink [71], which enrolled participants from primary care into a 6-week program through a Web-based account. Our intervention is designed to promote the take-up of other resources for longer-term engagement, where required. Effective engagement with such resources (eg, a mobile phone drink tracker) is likely to constitute a key mediator of the intervention’s effect.

### Limitations

This prototype intervention was developed in a single site in Southampton, United Kingdom. Feasibility and acceptability remain to be demonstrated in other sites, with different population demographics. The proposed intervention is also designed around the characteristics of the UK cancer detection model, and it may require adaptation to other health systems.

### Conclusions

Breast cancer is the most common type of cancer in women, and alcohol is one of the most feasible risk factors to moderate for the prevention of breast cancer [3]. Symptomatic breast clinics constitute a context in which targeted health improvement interventions could take place. Unlike other ABIs, the proposed intervention aims to be acceptable and feasible to deliver to all women who attend symptomatic breast clinics, irrespective of their level of alcohol consumption. Despite extensive research on ABIs, current evidence is predominantly restricted to increased-risk drinkers. It also provides little data on the maintenance of the effects of digitally delivered ABIs beyond 12 months [13]. The effectiveness of the proposed intervention thus requires further research.

## Appendix

Multimedia Appendix 1 Scope of literature reviews.

Multimedia Appendix 2 Archive of five views of the prototype web application.

## Abbreviations

6SQuID: 6 steps in quality intervention development
ABI: alcohol brief intervention
BCT: behavior change technique
NHS: National Health Service
NIHR: National Institute for Health Research
