# Supplementary material for: A Context-Specific Digital Alcohol Brief Intervention in Symptomatic Breast Clinics (Abreast of Health): Development and Usability Study
Source: JMIR Res Protoc. 2020 Jan 24;9(1):e14580. doi: 10.2196/14580 (PMC7007589; doi:10.2196/14580)
Supplement: Multimedia Appendix 1 [file resprot_v9i1e14580_app1.docx]

- **Review 1: Knowledge and social attitudes to alcohol.**

Google Scholar and snowball searches helped identify potentially relevant academic studies and grey literature covering alcohol use and at least one of the following: social attitudes; health beliefs and attitudes; health literacy; knowledge of effects of alcohol on health; readiness to change; self-efficacy; social networks and interpersonal determinants; other health lifestyles; health promotion marketing and segmentation; health inequalities. The review only considered references reporting findings on adult women and/or health care staff, with priority given to UK-based evidence.

- **Review 2: Social attitudes in relation to modifiable risk factors for cancer.**

Google Scholar and snowball searches helped identify potentially relevant academic studies and grey literature covering cancer and at least one of the following: social attitudes; health beliefs and attitudes; public understanding of risks; knowledge of cancer risk factors; information needs and information-seeking behaviours; cancer screening take-up; reaction to cancer information pamphlet and campaigns.

- **Review 3: Theories and reviews of behaviour change mechanisms and techniques.**
  - Cochrane library

Kaner EF, Beyer FR, Muirhead C, Campbell F, Pienaar ED, Bertholet N, Daeppen JB, Saunders JB, Burnand B. Effectiveness of brief alcohol interventions in primary care populations. Cochrane Database Syst Rev [Internet] 2018 Feb 24;(12). [doi: 10.1002/14651858.CD004148.pub4]

Kaner EF, Beyer FR, Garnett C, Crane D, Brown J, Muirhead C, Redmore J, O’Donnell A, Newham JJ, de Vocht F, Hickman M, Brown H, Maniatopoulos G, Michie S. Personalised digital interventions for reducing hazardous and harmful alcohol consumption in community-dwelling populations. Cochrane Database Syst Rev [Internet] 2017 Sep 25; [doi: 10.1002/14651858.CD011479.pub2]

- - Narrative and systematic reviews of alcohol interventions

Beyer F, Lynch E, Kaner E. Brief Interventions in Primary Care: an Evidence Overview of Practitioner and Digital Intervention Programmes. Curr Addict Reports [Internet] Current Addiction Reports; 2018 Jun 3;5(2):265–273. [doi: 10.1007/s40429-018-0198-7]

Black N, Mullan B, Sharpe L. Computer-Delivered Interventions for Reducing Alcohol Consumption: Meta-Analysis and Meta-Regression using Behaviour Change Techniques and Theory. Health Psychol Rev [Internet] Taylor & Francis; 2016;7199(April):1–33. PMID:26999311

Crane D, Garnett C, Brown J, West R, Michie S. Behavior Change Techniques in Popular Alcohol Reduction Apps: Content Analysis. J Med Internet Res [Internet] 2015 May 14;17(5):e118. [doi: 10.2196/jmir.4060]

Gaume, J., McCambridge, J., Bertholet, N., & Daeppen, J.-B. Mechanisms of Action of Brief Alcohol Interventions Remain Largely Unknown – A Narrative Review. Frontiers in Psychiatry. 2014;5(AUG):1–9. [doi: 10.3389/fpsyt.2014.00108]

Heather, N. Toward an Understanding of the Effective Mechanisms of Alcohol Brief Interventions. Alcoholism: Clinical and Experimental Research. 2014;38(3):626–628. [doi: 10.1111/acer.12336]

Johnson M, Jackson R, Guillaume L, Meier P, Goyder E. Barriers and facilitators to implementing screening and brief intervention for alcohol misuse: a systematic review of qualitative evidence. 2010;33(3):412–421. [doi: 10.1093/pubmed/fdq095]

Michie S, Whittington C, Hamoudi Z, Zarnani F, Tober G, West R. Identification of behaviour change techniques to reduce excessive alcohol consumption. Addiction [Internet] 2012 Aug;107(8):1431–1440. [doi: 10.1111/j.1360-0443.2012.03845.x]

O’Donnell, A., Wallace, P., & Kaner, E. From Efficacy to Effectiveness and Beyond: What Next for Brief Interventions in Primary Care? Frontiers in Psychiatry. 2014;5(August):113. [doi: 10.3389/fpsyt.2014.00113]

- - Systematic reviews of mechanisms of change and behaviour change techniques across behavioural science

Carey RN, Connell LE, Johnston M, Rothman AJ, de Bruin M, Kelly MP, Michie S. Behavior Change Techniques and Their Mechanisms of Action: A Synthesis of Links Described in Published Intervention Literature. Ann Behav Med [Internet] 2018 Oct 10; [doi: 10.1093/abm/kay078]

Cane J, O’Connor D, Michie S. Validation of the theoretical domains framework for use in behaviour change and implementation research. Implement Sci [Internet] 2012 Dec 24;7(1):37. [doi: 10.1186/1748-5908-7-37]

Michie S, Richardson M, Johnston M, Abraham C, Francis J, Hardeman W, Eccles MP, Cane J, Wood CE. The behavior change technique taxonomy (v1) of 93 hierarchically clustered techniques: Building an international consensus for the reporting of behavior change interventions. Ann Behav Med 2013;46(1):81–95. PMID:23512568
