# Supplementary material for: A Context-Specific Digital Alcohol Brief Intervention in Symptomatic Breast Clinics (Abreast of Health): Development and Usability Study
Source: JMIR Res Protoc. 2020 Jan 24;9(1):e14580. doi: 10.2196/14580 (PMC7007589; doi:10.2196/14580)
Supplement: Multimedia Appendix 2 [file resprot_v9i1e14580_app2.zip › Web capture/Breast cancer and alcohol/Breast cancer and alcohol.html]

Abreast of Health


Abreast of Health

# Breast Cancer and Alcohol

---

##### Research shows that regularly drinking alcohol is associated with a higher risk of developing breast cancer.

- Your risk of breast cancer will depend on how much alcohol you tend to drink.
- The more alcohol you regularly drink, the more likely you are to develop breast cancer at some point in your life.
- Even drinking just two units of alcohol a day can increase risk.

##### Breast cancer increases with the number of units of alcohol drunk per day

- Out of 100 women who do not drink, 11 will probably develop breast cancer in their lifetime.
- Out of 100 women who drink 2 units of alcohol a day (for example, a standard glass of wine), about 14 will develop breast cancer in their lifetime.
- In other words, drinking 2 units of alcohol a day results in 3 extra women out of 100 developing breast cancer.

  

See how your drink measures up

Tips and tricks on cutting down

  

- **Source:** Breast Cancer Now
- **Information last reviewed:** November 2017
- Adapted with thanks to Breast Cancer Now.

---

Home

##### How is this page?
