# Supplementary material for: A Context-Specific Digital Alcohol Brief Intervention in Symptomatic Breast Clinics (Abreast of Health): Development and Usability Study
Source: JMIR Res Protoc. 2020 Jan 24;9(1):e14580. doi: 10.2196/14580 (PMC7007589; doi:10.2196/14580)
Supplement: Multimedia Appendix 2 [file resprot_v9i1e14580_app2.zip › Web capture/Change my risks/Change my risks.html]

Abreast of Health


Abreast of Health

# Can I Change my Risks?

---

##### There are many different things that affect a woman’s risk of developing breast cancer.

  

Things you **cannot** change


Things you **can** change

  

### Things you cannot change

---

Some of these factors are difficult or not possible to change. These include:

- **Being female:** Women are much more likely to develop breast cancer than men.
- **Age:** Older women have a higher chance of developing breast cancer than younger women.
  The NHS invites women aged 47 to 73 years to attend
  breast screening
  once every three years. Those above the age of 73 years can self-refer to the program through their GP.
- **Life events:** Starting your periods at an older age, having children, and breastfeeding are all linked to a lower risk of developing breast cancer later in life.
- **Family history and genes:** Some women (around 15% of all women who develop breast cancer) have a strong family history
  of breast cancer and approximately 5% of women with breast cancer have inherited a change in a particular gene that means that they have
  a high risk of developing this disease. You can read more about this on
  Breast Cancer Now's website.
  If you think you may have a strong
  family history of breast cancer, or believe you are at increased risk, then please do talk to your GP about your concern.
- **Hormone therapies:** Taking hormone replacement therapy (HRT) or the contraceptive pill can also affect your risk of developing breast cancer. If this is something you are worried about we recommend that you discuss this with your GP.

### Things you can change

---

Although there are risk factors for breast cancer which you cannot change, you can reduce your risk of breast cancer by
paying attention to your lifestyle.

- Researchers estimate that 19% of breast cancers in the UK can be attributed to
  **physical inactivity**, **being overweight**, and **drinking alcohol**.
- **Alcohol** alone is thought to be responsible for **7% of breast cancers in the UK**
  and may in fact be **one of the easier risk factors to change**.

Press the buttons below to find out more about how these factors affect breast cancer risk and how you can reduce your risks.

Breast cancer risk and   
 body weight

Breast cancer risk and   
 alcohol

Breast cancer risk and   
 physical activity

  
Source: Cancer Research UK, Breast cancer risk factors, Accessed May 2018.

  


---

Home

##### How is this page?
