# Supplementary material for: A Context-Specific Digital Alcohol Brief Intervention in Symptomatic Breast Clinics (Abreast of Health): Development and Usability Study
Source: JMIR Res Protoc. 2020 Jan 24;9(1):e14580. doi: 10.2196/14580 (PMC7007589; doi:10.2196/14580)
Supplement: Multimedia Appendix 2 [file resprot_v9i1e14580_app2.zip › Web capture/Drink calculator/What's in your drink.html]

Abreast of Health


Abreast of Health

# What’s in your drink?

---

#### Step 1: Select a drink

#### Step 2: Enter quantity

###

Half

Add drink

---

#### Step 3: Check the total

Units

---

0.0

Calories

---

0.0

Food equivalent

---

0.0

Reset

---

## Units of alcohol

A UK unit is worth:

- 10 millilitres or
- 8 grams of pure alcohol.

It is also sometimes called a 'standard drink'.

## The doctors' recommendation

The Department of Health recommends that you:

- drink under 14 units a week
- keep at least 2 days free of alcohol each week
- never drink more than 6 units in a single session.

##### How is this page?
