# Supplementary material for: A Context-Specific Digital Alcohol Brief Intervention in Symptomatic Breast Clinics (Abreast of Health): Development and Usability Study
Source: JMIR Res Protoc. 2020 Jan 24;9(1):e14580. doi: 10.2196/14580 (PMC7007589; doi:10.2196/14580)
Supplement: Multimedia Appendix 2 [file resprot_v9i1e14580_app2.zip › Web capture/Improving your health and wellbeing/Improving your health and wellbeing.html]

Abreast of Health


Abreast of Health

# Improving my health and wellbeing

---

**Tap on the bubble icons to find out more.**

!


Improve your moodand reduce anxiety
Better focus andlower risks of dementia
A healthy liver
Improve your sleep
Protect your heartand circulation
Healthy digestionand low risks ofcancer
Staying safe


Nutrition


Healthy skin, hair and nails

  

×

## Improve Your Sleep

  

People often think that drinking alcohol helps them get to sleep. It may help make you sleepy,
but research shows it stops you from sleeping deeply by disrupting your natural sleep cycle,
leaving you tired the next day.

Many people find that by reducing their alcohol consumption they sleep more deeply,
and feel refreshed in the morning.

×

## Improved nutrition

Alcohol is packed with calories that contain no nutritional value and can lower blood sugar levels making you feel hungry and more likely to overeat food that is high in sugar and fat.

See how many calories you can save by reducing the amount you drink.

×

## Improved digestion

In construction

×

## Better focus and lower risks of dementia

  

Under construction

×

## Protect your heart and circulation

  

Under construction

×

## A healthy liver

  

Under construction

×

## Healthy skin, hair and nails

  

Healthy skin, hair and nails can really improve how healthy you look.

Alcohol dehydrates the skin making it look dull and tired. It also dilates the facial blood vessels, the cause of spidery red thread veins. Many alcoholic drinks are also high in sugar, which can cause inflammation and insulin spikes, which lead to puffy skin.

Your hair and nails will also benefit from good levels of hydration, as regular alcohol can leave them dry and brittle.

If you are feeling thirsty, why not replace drinks containing alcohol with ones that hydrate the body instead?

×

## Stay safe

Drinking too much, too quickly, on any single occasion can increase your risk of accidents (including falls), impair your judgment so you may think something is safe when it isn’t, and result in you doing things (driving, having unsafe sex, starting an argument) which you know you would not choose to do if you were fully in control.

Perhaps you could set yourself a limit on how much you will drink to stay safe and in control of what you do and say?

×

## Improve your mood and reduce anxiety

Many people drink alcohol to relax and combat feelings of stress, but alcohol’s effect on the brain actually increases feelings of depression and anxiety over time, and reduces the likelihood of you using other ways (e.g walks, chatting with friends, reading) that help keep stress levels under control and built up your resilience to stress.

Why not make a list of 10 things, not involving alcohol you could do to reduce stress? Commit to trying at least one a week.

Infographic vector created by Freepik


---

For helpful tips and apps to improve your health and wellbeing  
visit www.nhs.uk/oneyou

Home

##### How is this page?
