# Supplementary material for: A Context-Specific Digital Alcohol Brief Intervention in Symptomatic Breast Clinics (Abreast of Health): Development and Usability Study
Source: JMIR Res Protoc. 2020 Jan 24;9(1):e14580. doi: 10.2196/14580 (PMC7007589; doi:10.2196/14580)
Supplement: Multimedia Appendix 2 [file resprot_v9i1e14580_app2.zip › Web capture/Personalised feedback/Personalised feedback.html]

Abreast of Health


Abreast of Health


- Home
  (current)
- My Data

# Your Feedback

  

## Your Alcohol Intake

---

##### According to the data you just provided, you may be drinking about 14 units of alcohol per week, on average.

Your answers suggest you could reduce your chances of disease and improve your future health by making a few changes to your drinking.

Drinking little or no alcohol brings many health benefits, including keeping risks of breast cancer low.

  

##### What do you drink compared to others?

---

64%

67%

64
percent of women drink less alcohol

67
percent of women drink less alcohol

in this clinic
in England

##### To maintain good health, the Department of Health recommends to:

---

- Not drink more than 14 units of alcohol a week.
- Not drink 6 units or more on a single occasion .
- Have several alcohol-free days each week.

Find out what is in your drink

##### Risks of alcohol-related harm

---

## Your Smoking

---

- You said you do not smoke.
- Not smoking is one of the best ways to stay healthy.

## Weight

---

- Based on your answers, your body mass index (BMI) is 16.4.
  A BMI below 18.5 could mean your weight is a little low.
- If you are concerned about this, you can seek further advice from your GP.

---

Home
