# Supplementary figures and images for: A Context-Specific Digital Alcohol Brief Intervention in Symptomatic Breast Clinics (Abreast of Health): Development and Usability Study
Source: JMIR Res Protoc. 2020 Jan 24;9(1):e14580. doi: 10.2196/14580 (PMC7007589; doi:10.2196/14580)

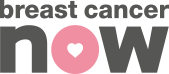

Supplement: Multimedia Appendix 2 [file resprot_v9i1e14580_app2.zip › Web capture/Breast cancer and alcohol/Breast cancer and alcohol_files/bcn_logo.png]

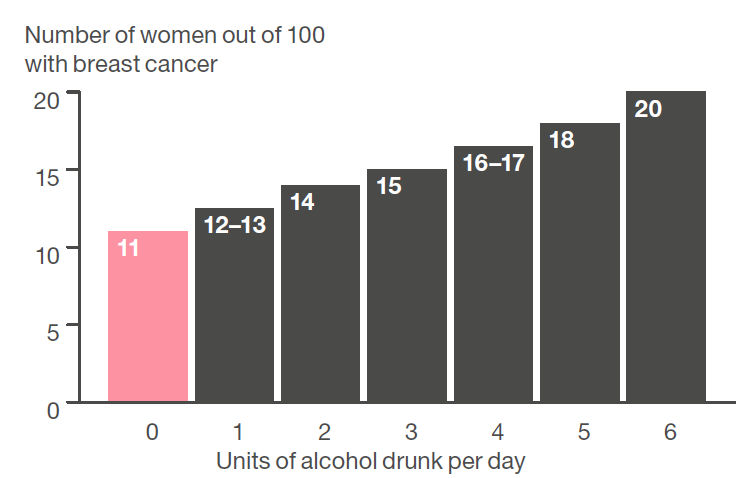

Supplement: Multimedia Appendix 2 [file resprot_v9i1e14580_app2.zip › Web capture/Breast cancer and alcohol/Breast cancer and alcohol_files/doseresponse.png]

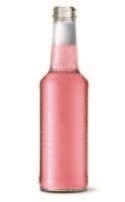

Supplement: Multimedia Appendix 2 [file resprot_v9i1e14580_app2.zip › Web capture/Drink calculator/What's in your drink_files/alcopop_275ml.jpg]

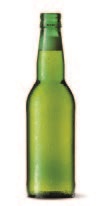

Supplement: Multimedia Appendix 2 [file resprot_v9i1e14580_app2.zip › Web capture/Drink calculator/What's in your drink_files/beerbottle_330ml.jpg]

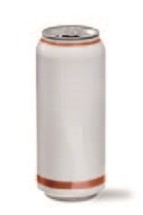

Supplement: Multimedia Appendix 2 [file resprot_v9i1e14580_app2.zip › Web capture/Drink calculator/What's in your drink_files/beercan_440ml.jpg]

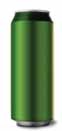

Supplement: Multimedia Appendix 2 [file resprot_v9i1e14580_app2.zip › Web capture/Drink calculator/What's in your drink_files/beercan_500ml.jpg]

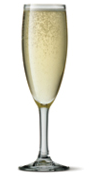

Supplement: Multimedia Appendix 2 [file resprot_v9i1e14580_app2.zip › Web capture/Drink calculator/What's in your drink_files/champagne.png]

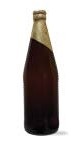

Supplement: Multimedia Appendix 2 [file resprot_v9i1e14580_app2.zip › Web capture/Drink calculator/What's in your drink_files/cider_500ml.jpg]

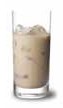

Supplement: Multimedia Appendix 2 [file resprot_v9i1e14580_app2.zip › Web capture/Drink calculator/What's in your drink_files/creamliqueur.jpg]

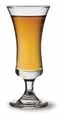

Supplement: Multimedia Appendix 2 [file resprot_v9i1e14580_app2.zip › Web capture/Drink calculator/What's in your drink_files/fortified_wine.jpg]

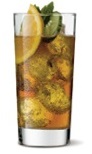

Supplement: Multimedia Appendix 2 [file resprot_v9i1e14580_app2.zip › Web capture/Drink calculator/What's in your drink_files/pimms.jpg]

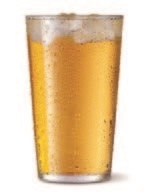

Supplement: Multimedia Appendix 2 [file resprot_v9i1e14580_app2.zip › Web capture/Drink calculator/What's in your drink_files/pint_A.jpg]

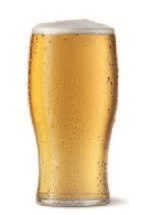

Supplement: Multimedia Appendix 2 [file resprot_v9i1e14580_app2.zip › Web capture/Drink calculator/What's in your drink_files/pint_B.jpg]

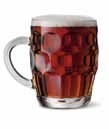

Supplement: Multimedia Appendix 2 [file resprot_v9i1e14580_app2.zip › Web capture/Drink calculator/What's in your drink_files/pint_C.jpg]

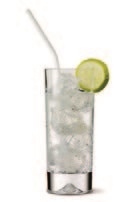

Supplement: Multimedia Appendix 2 [file resprot_v9i1e14580_app2.zip › Web capture/Drink calculator/What's in your drink_files/spiritsmallglass.jpg]

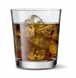

Supplement: Multimedia Appendix 2 [file resprot_v9i1e14580_app2.zip › Web capture/Drink calculator/What's in your drink_files/spirit_double.jpg]

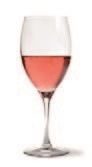

Supplement: Multimedia Appendix 2 [file resprot_v9i1e14580_app2.zip › Web capture/Drink calculator/What's in your drink_files/wine_125ml.jpg]

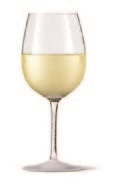

Supplement: Multimedia Appendix 2 [file resprot_v9i1e14580_app2.zip › Web capture/Drink calculator/What's in your drink_files/wine_175ml.jpg]

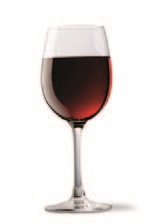

Supplement: Multimedia Appendix 2 [file resprot_v9i1e14580_app2.zip › Web capture/Drink calculator/What's in your drink_files/wine_250ml.jpg]

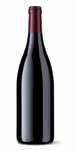

Supplement: Multimedia Appendix 2 [file resprot_v9i1e14580_app2.zip › Web capture/Drink calculator/What's in your drink_files/wine_bottle_750ml.jpg]

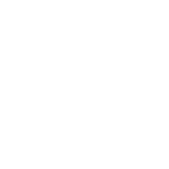

Supplement: Multimedia Appendix 2 [file resprot_v9i1e14580_app2.zip › Web capture/Personalised feedback/Personalised feedback_files/data.png]

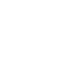

Supplement: Multimedia Appendix 2 [file resprot_v9i1e14580_app2.zip › Web capture/Personalised feedback/Personalised feedback_files/home.png]

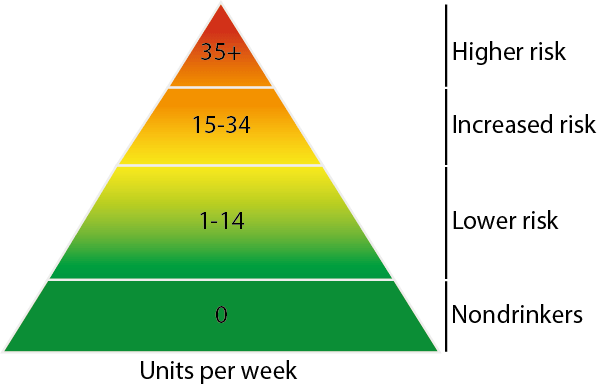

Supplement: Multimedia Appendix 2 [file resprot_v9i1e14580_app2.zip › Web capture/Personalised feedback/Personalised feedback_files/pyramid1_gradient.png]
